# Silica nanoparticles as pesticide against insects of different feeding types and their non-target attraction of predators

**DOI:** 10.1038/s41598-021-93518-9

**Published:** 2021-07-14

**Authors:** Ahmed F. Thabet, Hessien A. Boraei, Ola A. Galal, Magdy F. M. El-Samahy, Kareem M. Mousa, Yao Z. Zhang, Midori Tuda, Eman A. Helmy, Jian Wen, Tsubasa Nozaki

**Affiliations:** 1 Economic Entomology Department, Faculty of Agriculture, Kafrelsheikh University, Kafr El-sheikh, Egypt; 2 Genetics Department, Faculty of Agriculture, Kafrelsheikh University, Kafr El-sheikh, Egypt; 3 Field Crop Pests Research Department, Plant Protection Research Institute, Agricultural Research Center, Sakha, Kafr El-sheikh, Egypt; 4 Laboratory of Insect Natural Enemies, Institute of Biological Control, Department of Bioresource Sciences, Faculty of Agriculture, Kyushu University, Fukuoka, 819-0395 Japan; 5 Regional Centre for Mycology and Biotechnology (RCMB), Al-Azhar University, Cairo, Egypt; 6 Entomological Laboratory, Graduate School of Bioresource and Bioenvironmental Sciences, Kyushu University, Fukuoka, Japan; 7 The Kyushu University Museum, Fukuoka, Japan

**Keywords:** Nanobiotechnology, Behavioural methods, Population dynamics

## Abstract

The agricultural use of silica (SiO_2_) nanoparticles (NPs) has the potential to control insect pests while the safety and tritrophic effects on plants and beneficial natural enemies remains unknown. Here, we evaluate the effects of silica NPs on insect pests with different feeding niches, natural enemies, and a plant. Silica NPs were applied at different concentrations (75–425 mg/L) on field-cultivated faba bean and soybean for two growing seasons. The faba bean pests, the cowpea aphid *Aphis craccivora* and the American serpentine leafminer *Liriomyza trifolii*, and the soybean pest, the cotton leafworm *Spodoptera littoralis*, were monitored along with their associated predators. Additional laboratory experiments were performed to test the effects of silica NPs on the growth of faba bean seedlings and to determine whether the rove beetle *Paederus fuscipes* is attracted to cotton leafworm-infested soybean treated with silica NPs. In the field experiments, silica NPs reduced the populations of all three insect pests and their associated predators, including rove beetles, as the concentration of silica NPs increased. In soybean fields, however, the total number of predators initially increased after applying the lowest concentration. An olfactometer-based choice test found that rove beetles were more likely to move towards an herbivore-infested plant treated with silica NPs than to a water-treated control, suggesting that silica NPs enhance the attraction of natural enemies via herbivore-induced plant volatiles. In the laboratory, while silica NPs inhibited the development of faba bean roots at 400 mg/L, they did not affect germination percentage, germination time, shoot length, or vigor index compared to the control.

## Introduction

Nanotechnology is one of the most important tools in modern agriculture and is expected to revolutionize the field of pest management in the near future^1^. Nanoparticles (1–100 nm in diameter) can be used as effective pesticides against weeds, plant pathogens, and insect pests and may also be incorporated into new formulations of insecticides and insect repellents^2–4^ without the hazards posed by traditional chemical pesticides on beneficial insects (such as natural enemies)^5^, public health, and the environment^6^. Unlike conventional hydrophobic pesticides, nanocides may be water-soluble (hydrophilic), which enhances bioactivity and coverage uniformity^7^. Because they can be applied in small volumes and are taken in quickly by cells, the use of nanocides can slow the development of resistance in target pests^8^.

Among nanomaterials, silica (SiO_2_) nanoparticles (NPs) have received considerable attention as a possible alternative to conventional insecticides. The insecticidal properties of silica NPs are thought to be due to direct abrasion of the insect cuticle^8–10^ or sorption through the cuticular layers^2^. Silica NPs may also have an indirect insecticidal effect on pests feeding on treated plants or food by blocking the digestive tract^11^ and inducing malformation of external morphology (Thabet et al. unpublished). Silica may also impair the digestive tract in insect herbivores that feed on silica-treated plants^12^. The direct effects of silica NPs on a variety of pest insects have been examined in the lab (e.g. Mousa et al.^13^; Supplementary Table S1). The in-field effects of silica NPs have been evaluated for a limited number of insect pests and they are negative and dose-dependent; evaluated species include a chewing insect [moth: *Spodoptera littoralis*^14^], a piercing-sucking insect [aphid: *Aphis craccivora*^15^], and an internal feeder [leafmining fly: *Liriomyza trifolii*^15^] (Supplementary Table S1).

In tritrophic food chains, plants (crops), herbivores (insect pests), and their predators and/or parasitoids (natural enemies) are influenced by direct and indirect interactions with each other. At the base of these food chains, plants uptake silicon (Si, bulk size > 1 μm) from the soil, and their ability to do so varies according to different mechanisms of uptake^16,17^. Si absorbed and deposited in plant epidermal tissue (cell walls, lumen, intracellular space, and trichomes/hairs)^18^ as amorphous hydrated silica (SiO_2_·nH_2_O) can increase the rigidity of the tissue, causing increased wear to the mandibles of chewing insects^19–25^ and modification of the mouthparts of dipteran larvae^11^, along with increased alleviation of environmental stress on plants^26^. Moreover, Si enrichment in plants biochemically defends against herbivores, by priming for jasmonate-mediated inducible defenses (with defense-related enzymes and proteins)^27^. It may also enhance and/or alter herbivore-induced plant volatiles (HIPVs) emitted from infested plants, which ultimately direct natural enemies (predators or parasitoids) to the prey or hosts that infested the plants^28^. Plant volatiles also can alert nearby undamaged plants of impending risk, and travel within the plant system as pest feeding/oviposition deterrents^29^. Therefore, Si accumulation in plants results in a cascade effect on the attraction of natural enemies of pests that use plant volatile cues to locate their prey or hosts^30^. The role of Si in attracting natural enemies by enhancing hormonal signaling has been known in Si-accumulators, such as rice^30–33^ (e.g. against the leaffolder *Cnaphalocris medinalis* by attracting the parasitoids *Trathala flavororbitalis* and *Microplitis mediator*^34^). Silicon supplementation has also been found to decrease herbivores in medium Si-accumulators or non-accumulators, namely, soybean (against *Helicoverpa punctigera*)^35^, cucumber (against the cotton bollworm *Helicoverpa armigera* by attracting the predatory beetle *Dicranolaius bellulus*)^36^, and grapevine (against the light brown apple moth *Epiphyas postvittana* by attracting *D. bellulus*)^37^. Whether nanoscale silicon (or nanosilica) has a similar indirect tritrophic effect as bulk silicon is currently unknown.

The known effects of silica NPs are limited. However, their effects on the predators *Coccinella* spp., *Chrysoperla carnea*, and true spiders in the field are negative^14^, which might include an indirect effect of poor quality of their prey that were directly impacted by silica NPs. As for bulk silica and Si, their effects on predators or parasitoids are neutral^5,38^ or negative via prey that fed on Si-treated plants^38^ (Supplementary Table S2).

The direct effects of silica NPs on plant growth may be positive^38–43^, non-significant^44,45^ or negative^45–48^ (Supplementary Table S3). On faba bean seedlings, silica NPs have concentration-dependent genotoxicity but no effects on growth in the limited concentration range of 25–75 mg/L^44,49^. On soybean yield in the field, silica NPs increased the number of pods and seeds, seed weight, and total yield dose-dependently in the range of 250–450 mg/L^50^.

The cotton leafworm *Spodoptera littoralis* is a tropical and subtropical destructive invader with a diverse host plant range spanning at least 87 economically significant species belonging to more than 40 families^51^. This A2 quarantine (locally present in European and Mediterranean regions) noctuid pest^52^ has developed a high resistance to chemical and biological pesticides. The late-instar larvae vigorously feed leaves, flower buds, and bolls^53^. The tropical small aphid, the cowpea aphid *Aphis craccivora*, is a polyphagous pest with a strong preference for Fabaceae and is capable of rapid population growth^54^ by the successive production of apterous adult females in conditions of sufficient food and favorable habitat^55^. Heavy feeding by both adults and nymphs often kills young plants while also causing leaf distortion, flowering delay, and a drop in fruit yield^56^ because their honeydew enhances mold formation^57^ and their feeding spreads viral infections among plants^58^. The American serpentine leafminer *Liriomyza trifolii*, is a devastating pest of faba bean (*Vicia faba*) and other crops^59,60^. Each of these pests represents a unique feeding niche: *A. craccivora* inhabit the external surfaces of plants and use their piercing-sucking mouthparts to feed on phloem; *L. trifolii* larvae live within the leaf tissues and feed internally; and *S. littoralis* larvae inhabit on the external surfaces of plants, chewing plant tissues. Because of these niche differences, each pest may be affected by silica NPs in a different way.

Lady beetles (Coccinellidae), rove beetles (*Paederus*), lacewings (*Chrysoperla*), and minute pirate bug (*Orius*) are the dominant generalist predator species conjugated in biological control strategies of the above-mentioned insect pests^60–66^. Soybean (*Glycine max*) is the most important crop among fabaceous plants as a protein-rich food source (including meat substitutes) and livestock feed. Faba bean (*Vicia faba*) is one of the most important carbohydrate food sources and is also used as livestock feed and fodder.

Here, we test the following hypotheses to evaluate the effect of silica NPs on tritrophic agricultural systems, in which the pests have different feeding niches: Silica NPs sprayed on leaves (1) negatively affect pest populations dose-dependently, (2) affect internal feeders less than external feeders, (3) either negatively or positively affect predator populations dose-dependently, and (4) negatively affect plant germination dose-dependently. Our study system comprises the major insect pests of faba bean (the cowpea aphid *Aphis craccivora* and the American serpentine leafminer *Liriomyza trifolii*) and soybean (the cotton leafworm *Spodoptera littoralis*).

## Materials and methods

### In-field effects of silica NPs on *A. craccivora* and *L. trifolii* on faba bean

**Figure 1:**
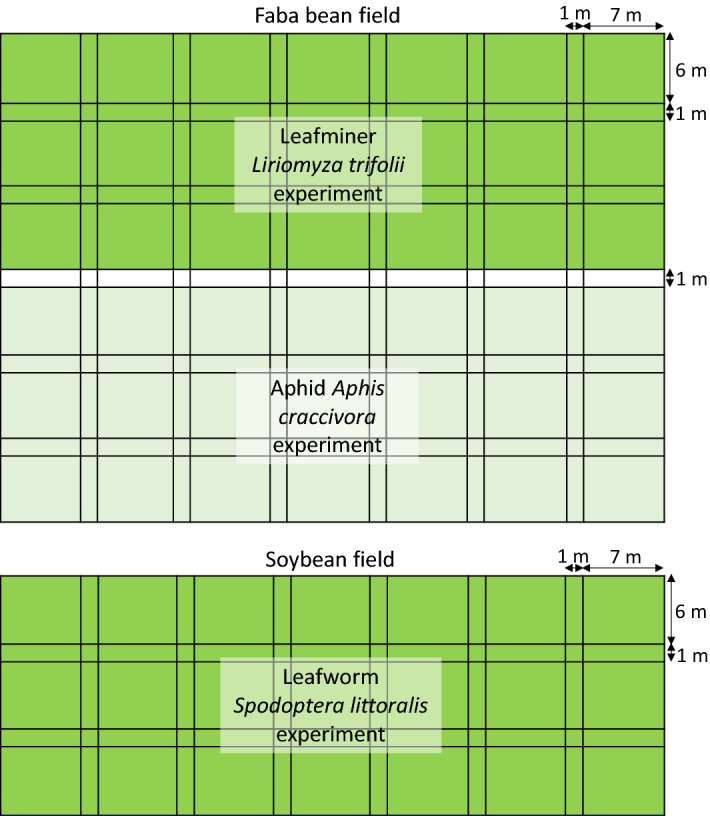
A schematic figure of the faba bean and soybean fields. Shared color of the field indicates the same location.

Faba bean (*V. faba*) seeds were obtained from Food Legumes Research Section, Sakha Agricultural Research Station, Egypt. Silica NPs with a 29-nm diameter and in the spherical form (hydrophilic, 99.99% purity) were purchased from NanoTech Egypt Co. (Dreamland, Egypt). Six different concentrations—75, 150, 225, 300, 375, and 425 mg/L—were chosen following Elsamahy and Galal^15^ and prepared following Thabet et al.^44^. The experimental field was divided into two sections: one for studying the efficacy of silica NPs against the cowpea aphid *A. craccivora* and another for studying their efficacy against the American serpentine leafminer *L. trifolii*. Two to three faba bean seeds per niche were sown 25 cm apart between niches on each of ten 6-m-long ridges in a 6 × 7 m area (a replicate), with three replications per treatment (i.e., NP concentration) (upper panel in Fig. 1) in a randomized complete block design set up in 2013 and 2014. Seeds were planted in mid-November of each year prior, and strips 1 m in width were left free from cultivation between neighboring replicates and treatments (upper panel in Fig. 1). Normal cultural practices were applied without the use of insecticides. Silica NPs (5 L per replicate) at each of the six different concentrations (three replicates per treatment), or distilled water as a control, were applied once in mid-March using ordinary knapsack sprayers with one atomizer, ensuring full coverage of the plants. These field experiments were carried out at the Experimental Farm and Field Crop Pests Research Laboratory of Plant Protection Research Institute (PPRI), Sakha Agricultural Research Station, Kafr El-Sheikh, Egypt. The abundance of live cowpea aphid *A. craccivora* or American serpentine leafminer *L. trifolii* was recorded for each replicate immediately before spraying as well as 1, 3, 5, 7, 10, and 15 days after spraying. The number of *A. craccivora* (nymphs and adults) was directly recorded in the field, and the number of *L. trifolii* larvae was counted by examining the leaf mines under a stereoscopic binocular microscope from a random sample of 25 leaflets per replicate. The percentage reduction in the size of the pest population was calculated using Henderson-Tilton's formula^67^ as follows:

1 $${\text{Reduction}}\;{\text{\% }} = \left[ {1 - \frac{{{\text{population}}\;{\text{size}}\;{\text{after}}\;{\text{treatment}} \times {\text{population}}\;{\text{size}}\;{\text{before}}\;{\text{control}}\;{\text{treatment}}}}{{{\text{population}}\;{\text{size}}\;{\text{before}}\;{\text{treatment}} \times {\text{population}}\;{\text{size}}\;{\text{after}}\;{\text{control}}\;{\text{treatment}}}}~} \right] \times 100$$

### In-field effects of silica NPs on *S. littoralis* on soybean

Following the same methodology as in the faba bean fields, three soybean seeds (also obtained from Food Legumes Research Section, Sakha Agricultural Research Station) were planted as described in the previous section (lower panel in Fig. 1). Three to four soybean seeds were sown per niche, with 15 cm between niches, during the first week of May in each of the two growing seasons (2013 and 2014). Normal cultural practices were applied without the use of insecticides. Randomly chosen plants were used to calculate infestation by the cotton leafworm *S. littoralis* larvae. When about 25% of plant leaves were consumed by the leafworm, either distilled water (as a control treatment) or silica NPs from one of the six different concentrations (5 L per replicate, three replicates per treatment) was applied to the soybean plants using an ordinary knapsack sprayer (as described in the previous section). Ten shoots per replicate were randomly chosen and shaken by hand into a white bucket to count live larvae immediately before spraying as well as after 1, 3, 5, 7, 10, and 15 days after spraying. The percentage reduction after spraying was calculated using the Henderson-Tilton formula.

### Effects of silica NPs on predators in the field

To collect predators from faba bean and soybean fields, a sample of 15 shoots was examined per replicate. Samples were taken immediately before spraying as well as 1, 3, 5, 7, 10, and 15 days after spraying. Shoots were kept in a black box [30 × 20 × 10 cm (length × width × height)] with a glass tube (1.5 cm in diameter, 20 cm in length) attached to a hole on each of the four sides of the box so the predators could move into the tubes towards natural light. The number of predators was recorded after 24 h, and the percentage reduction after spraying was calculated using the Henderson–Tilton formula.

### Influence of silica NPs on predator choice

To test whether silica NPs can attract predators via plant (soybean) volatiles induced by the cotton leafworm, we performed the following choice test. *Spodoptera litura*, closely related to *S. littoralis*^68^ was used in this experiment because *S. littoralis* was unavailable in Japan where the experiment took place.

#### Predator

Rove beetles *Paederus fuscipes* (Coleoptera: Staphylinidae) were collected from rice fields on the campus of Kyushu University (Fukuoka City, Japan) in late February and mid-October 2020. They were kept on rice plants in plastic pots (12 cm max diameter, 7 cm in height) filled with peat moss, and the pots were mounted in transparent plastic cylinders (11 cm in diameter, 20 cm in height) with the tops covered with muslin and held in a climate-controlled room (25 ℃, 50% RH, 16:8 h L:D). *Sciaridae* sp. (Diptera) as well as eggs and first-instar larvae of the cotton leafworm *S. litura* were provided as prey for the beetles. The beetles were deprived of prey 24 h prior to the choice experiment. Individual rove beetles were isolated in glass vials (1.3 cm in diameter, 4.5 cm in height) one hour before the experiment.

#### Infested plants

Soybean seeds were germinated in plastic pots (10 cm in diameter, two seeds per pot) filled with vermiculite in a climate-controlled room (25 ℃, 50% RH, 16:8 h L:D). The pots were kept in trays for bottom-watering, so water mixed with water-soluble fertilizer (N: P: K ratio = 6:10:5, no silicon, Hyponex, Hyponex Japan, Osaka) was added as needed. The unifoliate leaves of VC soybean plants (the two-leaf stage, approximately two weeks old) were inoculated with five first instar larvae of the cotton leafworm *S. litura* that were transferred from a laboratory culture using a soft brush. The laboratory culture was maintained on an artificial diet (Insecta LFS, Nosan Co., Yokohama, Japan).

#### Choice test

Two days after inoculating the soybean plants with the cotton leafworm *S. litura*, the leaves were sprayed with 2 ml of a 75 mg/L solution of silica NPs (US Research Nanomaterials, Inc., Houston, USA) or with 2 ml of distilled water. The concentration of silica NPs (75 mg/L) was chosen based on the field experimental result that showed an increase in the number of predatory individuals. Spraying was done at a distance of 15 cm from the plant leaves. The choice experiments were performed using a glass Y-tube olfactometer (3.6 cm in diameter, 18 cm central tube) two hours after spraying the plants. For each arm of the two arms of the olfactometer, an air compressor was used to pass air through activated charcoal granules for purification at a rate of 4 L/min before entering a glass vessel (8 cm in diameter) that contained an isolated soybean plant infested with *S. litura*. After removing the lid of its glass vial, each rove beetle was allowed 30 s to exit the vial and enter the olfactometer. One of the arms of the olfactometer was considered chosen if the beetle travelled upwind via the central tube and progressed at least 4 cm into that arm. We also recorded the amount of time required for the beetles to make their choice. Each beetle was used once and sexed after the experiment. A total of 41 beetles were tested (30 females and 11 males). A total of seven pairs of infested soybean plants (one plant treated with silica NPs and another treated with distilled water) were used in the experiment. Each pair of plants was used for testing multiple rove beetles consecutively and replaced with a new pair (five beetles in March, eight beetles on average in October, depending on the availability of rove beetles and plants of similar size and condition). Whenever the pair of plants were replaced with a new pair, the Y tube was cleaned with 99% ethanol, the connecting tubes were washed with distilled water, and the positions of the control and treated plants were switched. The experiment was conducted in a climate-controlled room (25 ℃, 50% RH, 16:8 h L:D).

### Effects of silica NPs on the germination and growth of *V. faba* seedlings

To investigate their potential toxicity on M_1_ (the first generation after treatment by a mutagen, silica NPs) *V. fab*a plants, silica NPs were prepared as follows. A white powder of silica NPs (99.5% purity) was obtained from US Research Nanomaterials, Inc. (Houston, Texas, USA). The morphology of the NPs was inspected using a high-resolution transmission electron microscope (TEM) (JEM-2100, JEOL Ltd.) at an accelerating voltage of 200 kV; the silica NPs were confirmed to be 19.6 ± 5.8 nm (mean ± SD) in size and spherical in shape. A stock solution (1000 mg/L) of silica NPs was prepared by dissolving the powder in distilled water. This solution was sonicated for 30 min and centrifuged (2000 rpm, 25 °C) for another 30 min to precipitate the non-dispersed agglomerated particles, which were filtered out of the solution using No. 2 filter paper (90 mm in diameter, Advantec, Japan). Then, four different concentrations of silica NPs—50, 100, 200 and 400 mg/L—were prepared.

Dry and healthy *V. faba* seeds [obtained from Canada by Kokusai Pet Food (Kobe, Japan)] were sanitized using a diluted sodium hypochlorite solution for 3 min and washed three to four times with distilled water before being soaked in distilled water for 3 h. Then, the seeds were immersed in one of the silica NP concentrations (60 seeds for each of the four concentrations plus a distilled water control) for 24 h. After treatment, the seeds were thoroughly washed with distilled water to remove any residual NPs to exclude potential effects of residuals^69^ that otherwise might disperse to the surrounding environment, alter microbial community, and be absorbed by germinating seedlings. To evaluate germination, NP-treated and control seeds were sown in six replicates for each treatment in a randomized complete block design; ten seeds per replicate were approximately equally spaced and allowed to germinate and grow in a Petri dish (six dishes per treatment, i.e., NP concentration) 15 cm in diameter and lined with cotton moistened with distilled water. Seeds were considered to have germinated when the radicle was at least 3 mm in length, and the number of germinated seeds was counted every morning. Final germination percentage (G%) and mean germination time (MGT) were assessed to detect the effects of silica NPs on germination. These measures were calculated following Edmond and Drapala^70^ and Ranal and de Santana^71^:

2 $${\text{G}}\% = \frac{{\sum {n_{i} } }}{N},$$

where $n_{i}$ is the number of germinated seeds on day *i* and *N* is the total number of seeds in the experimental treatment, and

3 $${\text{MGT}} = \frac{{\sum {n_{i} t_{i} } }}{{\sum {n_{i} } }}$$

where $t_{i}$ is the time in days from seeding to germination on day *i*.

In order to evaluate seedling growth, the seeds in each replicate were moved on the fifth day of the experiment into plastic pots filled with vermiculite and irrigated with water. The seeds were allowed to grow under laboratory conditions (25 ± 1 °C and 8L:16D). Seedling growth, in terms of shoot and root length, was measured after two weeks as the mean of five seedlings per replicate, with six replicates per treatment, i.e., NP concentration. The values of these two criteria were summed to calculate the seedling vigor index (SVI), which serves as a collective measure of growth and was calculated according to Dahindwal et al.^72^’s equation:

4 $${\text{SVI}} = {\text{G}}\% \times {\text{mean}}\;{\text{seedling}}\;{\text{length}}$$

All methods were carried out in accordance with relevant guidelines and regulations in Egypt and Japan. All experimental protocols were approved by Sakha Agricultural Research Station, Plant Protection Research Institute, Kafrelsheikh University and Kyushu University.

### Statistical analysis

A MANOVA with repeated measures was used to evaluate the percentage reduction over the first three days as a function of the following explanatory variables: year of experiment, concentration of silica NPs, plant species, insect species (for pests)/guilds (for predators) nested within plant species, and their interaction (silica NP concentration × insect [plant species], silica NP concentration × plant species). We confirmed the normality of the distributions of the residuals. A post hoc Bonferroni test was performed for significant categorical variables. Two-tailed *t* tests were performed to determine whether the percentage reduction in pests and predators differed from zero (the population size before treatment) at each interval of time (1, 3, 5, 7, 10, and 15 days) following the application of the different concentrations of silica NPs.

To evaluate predator choice, a binomial test was performed based on an equal probability (0.5) of choosing either water-treated or NP-treated plant volatiles. A logistic regression model was applied to test the effect of sex on choice. A parametric survival analysis with the best fit log-normal distribution was used to test the explanatory effects of treatment, sex, and season on the amount of time taken to make a choice, with plant pair as a random variable.

The effects of silica NPs on plant growth parameters (G%, SVI, root length, and shoot length) were analyzed with nonparametric Wilcoxon/Kruskal–Wallis tests, followed by post hoc Steel’s multiple comparison tests with the control. MGT was analyzed using a parametric survival test with the best fit Frechet distribution. All statistical tests were performed using JMP 14.2.0.

## Results

**Figure 2:**
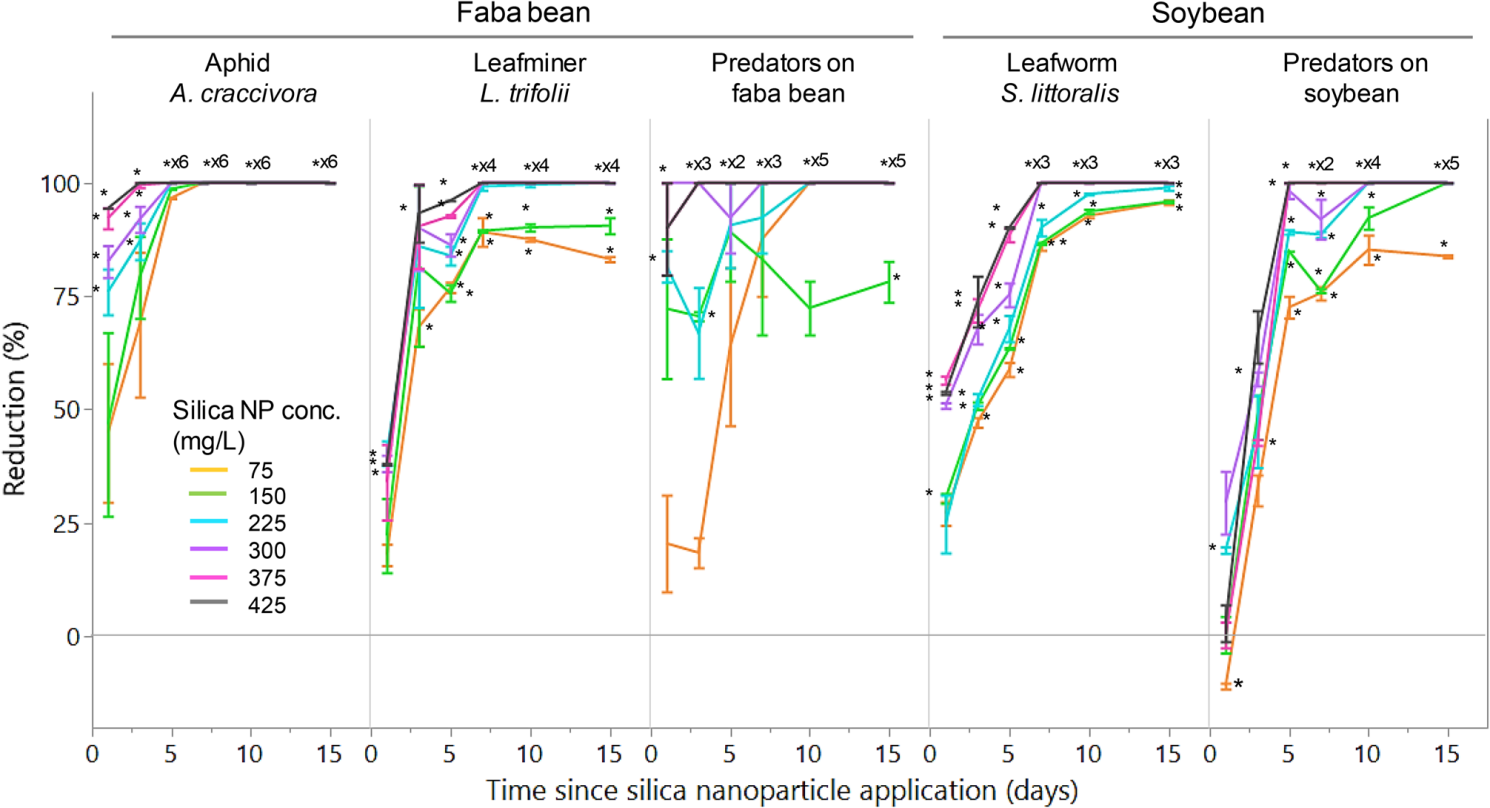
Reduction (mean ± SE %) over time in the population sizes (relative to the control) of pest insects (the cowpea aphid *Aphis craccivora*, the American serpentine leafminer *Liriomyza trifolii* and the cotton leafworm *Spodoptera littoralis*) and their insect predators 1–15 days after spraying different concentrations of silica (SiO_2_) nanoparticles on faba bean (*Vicia faba*) or soybean (*Glycine max*) in the field. An asterisk indicates a significant difference from zero (zero indicates no difference from the control). Overlapping means with an asterisk followed by multiplying numbers indicates the number of overlapping means with a significant difference from zero.

Irrespective of insect species or group, the reduction in population size relative to the control tended to saturate after five to seven days since applying the silica NPs (Fig. 2). Therefore, we subjected the data from the first 3 days following the silica NP application to statistical tests, confirming the normality of residuals distribution.

### Predator species in faba bean and soybean fields

Six species of predators were collected from NP-treated faba bean plants: *Paederus alfierii* (Coleoptera: Staphylinidae), *Coccinella undecimpunctata*, *Cydonia vicina isis, Cydonia vicina nilotica, Scymnus interruptus* (Coleoptera: Coccinellidae), and *Chrysoperla carnea* (Neuroptera: Chrysopidae). All six of these species were also detected on NP-treated soybean plants, with the addition of *Orius* spp. (Hemiptera: Anthocoridae).

### Reduction of pests and predators in faba bean and soybean fields

**Figure 3:**
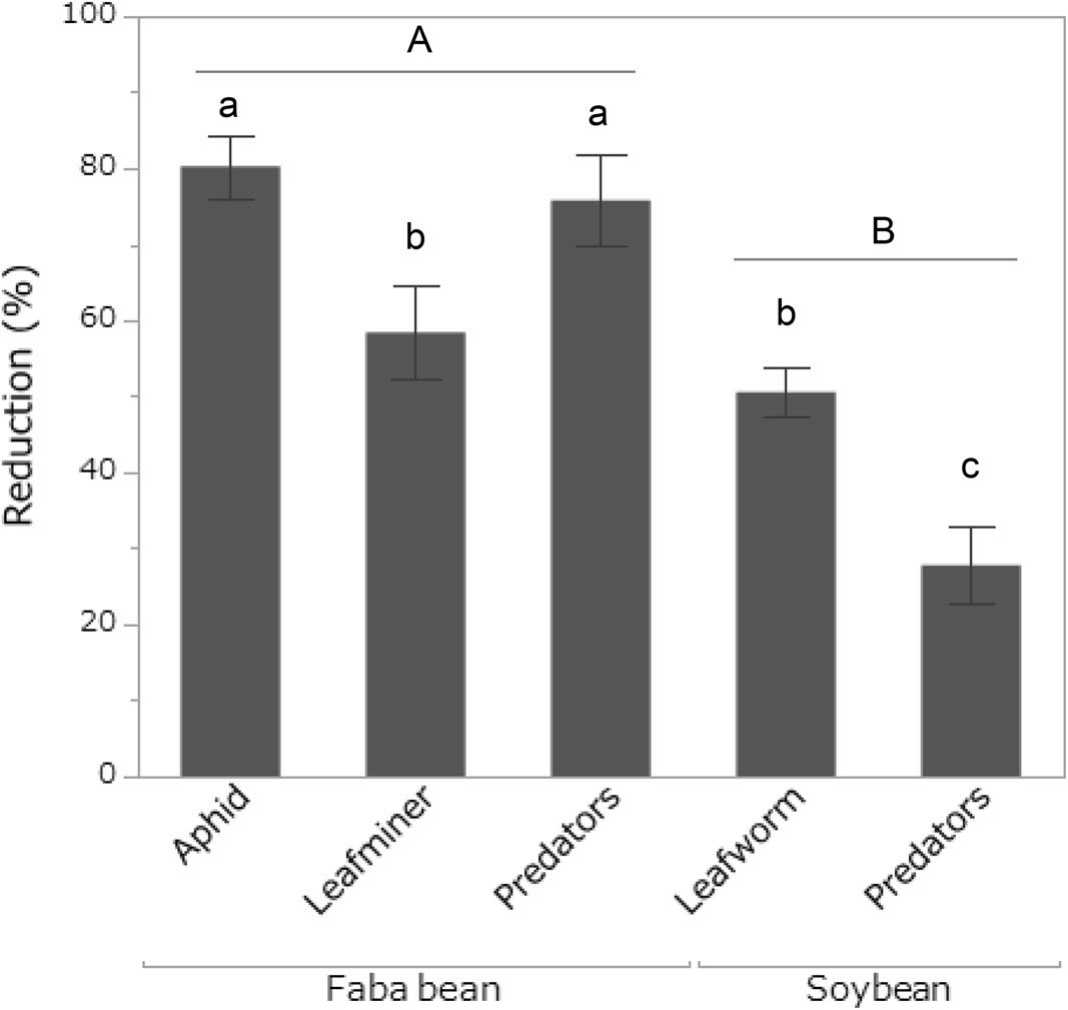
Reduction (mean ± SE %) in the population sizes of pest insects (the cowpea aphid *Aphis craccivora*, the American serpentine leafminer *Liriomyza trifolii* and the cotton leafworm *Spodoptera littoralis*) and their insect predators 1–3 days after spraying silica (SiO_2_) nanoparticles of different concentrations on either faba bean (*Vicia faba*) or soybean (*Glycine max*) in the field. Shared letters indicate no significant differences.

**Figure 4:**
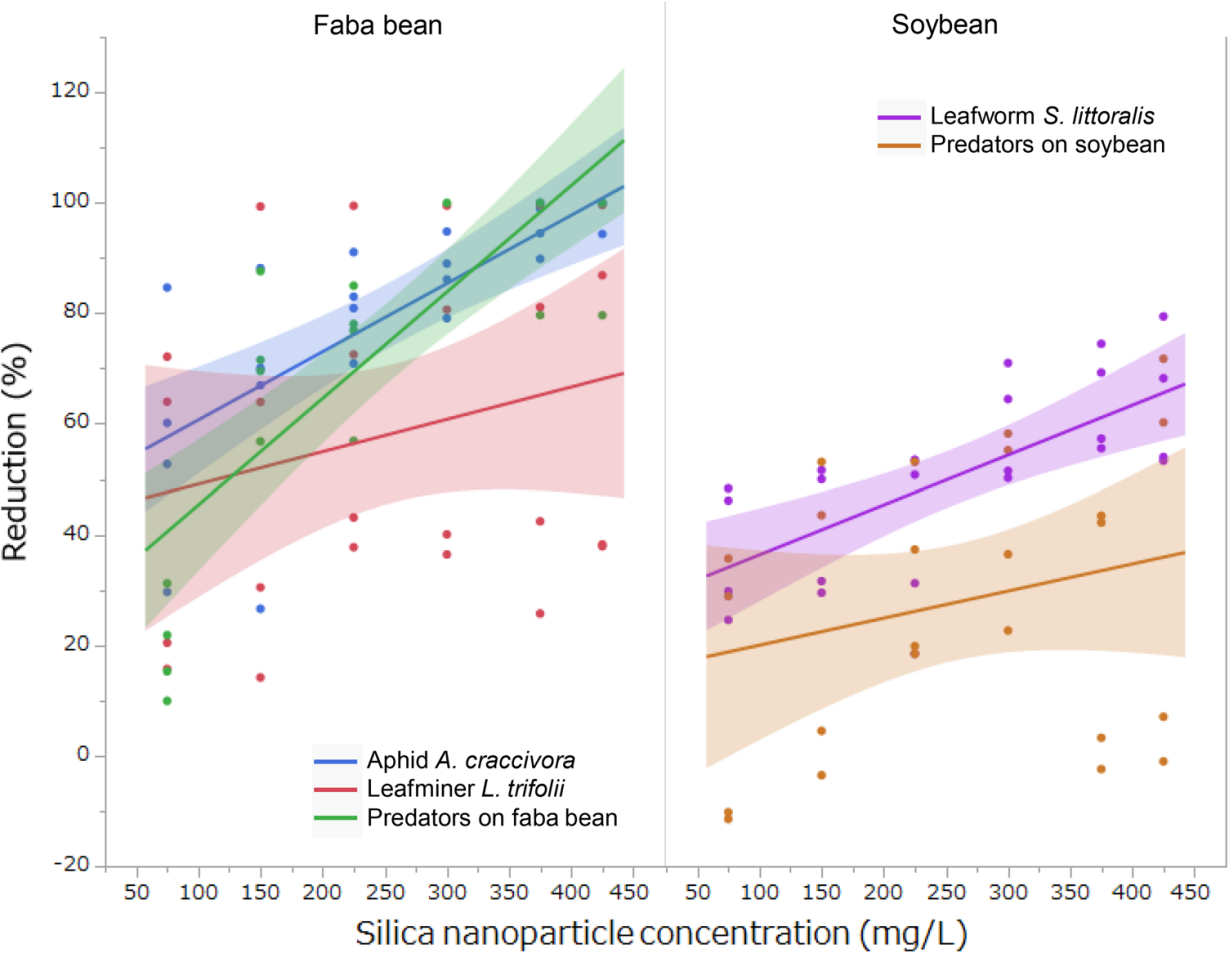
Relationship between silica (SiO_2_) nanoparticle concentration and the reduction of pest and predator insects 1–3 days after spraying silica nanoparticles on either faba bean (*Vicia faba*) or soybean (*Glycine max*) in the field. Shaded area indicates 95% confidence interval.

Population reductions increased with increasing concentrations of silica NPs, and the reductions were higher on faba bean than on soybean and differed among insect species/guilds nested within plant species (Table 1). The highest reductions were of the cowpea aphid *A. craccivora* and its predators on faba bean, followed by the American serpentine leafminer *L. trifolii* and the cotton leafworm *S. littoralis*, and the lowest reductions were in the predators of *S. littoralis* (Fig. 3). There was a significant interaction between insect species/guild nested within plant species and the concentration of silica NPs; slopes of concentration-dependent increases in percentage reduction were similar among *A. craccivora*, *L. trifolii*, *S. littoralis* and the predators in soybean, whereas it was notably steeper for predators on faba bean (Fig. 4). There was also a significant interaction between plant species and the concentration of silica NPs; the increase in reduction percentage according to the silica NP concentration was larger for insects on faba bean than for insects on soybean (Fig. 4). The effect of year was not significant (Table 1).

**Table 1:** MANOVA result on the reduction (%) of pest and predator insects on either faba bean or soybean plants in the field after 1–3 days of silica (SiO_2_) nanoparticle (NP) application.

Variable	Epsilon	*F*	Numerator df	Denominator df	*P*
Intercept	1.61	78.65	1	49	< 0.001
Silica NP concentration	1.36	66.40	1	49	< 0.001
Plant species	2.51	122.80	1	49	< 0.001
Insect species/guild [plant species]	1.06	17.26	3	49	< 0.001
Year	0.002	0.39	1	49	0.754
Insect species/guild [plant species] × silica NP concentration	0.29	4.81	3	49	0.005
Plant species × silica NP concentration	0.11	5.38	1	49	0.025

Reduction measured on different dates were treated as repeated measures. Insect species/guild [plant species]: insect species (pests) or guild of predators nested within plant species.

For the cowpea aphid *A. craccivora*, complete (100%) reduction was observed 5–15 days after applying silica NPs at concentrations of 225–425 mg/L and after 7–15 days at 75–150 mg/L (Fig. 2). Complete reductions in the American serpentine leafminer *L. trifolii* and the cotton leafworm *S. littoralis* occurred 7–15 days after applying silica NPs at 300–425 mg/L (Fig. 2). For predators on faba bean, complete reductions were observed after 3–15 days at 375–425 mg/L, after 7–15 days at 300 mg/L and after 10–15 days at 225 mg/L (Fig. 2). For predators on soybean, complete reductions were observed after 5–15 days at 375–425 mg/L, after 10–15 days at 225–300 mg/L and after 15 days at 150 mg/L (Fig. 2).

By contrast, the reduction was significantly negative (− 10.83 ± 0.87%, mean ± SD, *t* =  − 17.60, df = 1, *P* = 0.0361) for predators on soybean one day after applying silica NPs at 75 mg/L, the lowest concentration used in the experiment (Fig. 2). This indicates that the number of predators increased shortly after NP application.

### Influence of silica NPs on predator choice

**Figure 5:**
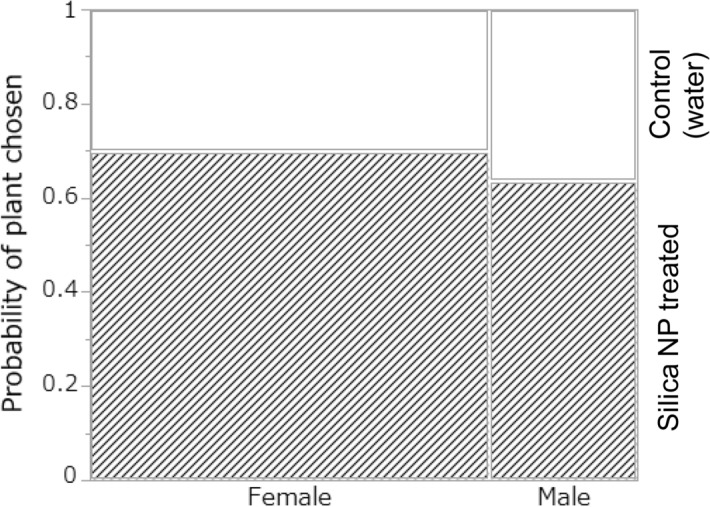
Probability of a plant being chosen by the rove beetle *Paederus fuscipes*. Shaded area: the probability of the beetle choosing an infested plant (soybean infested with the cotton leafworm *Spodoptera litura* larvae) treated with silica nanoparticles (NPs). White area: the probability of the beetle choosing an infested plant treated with water.

Rove beetles preferred *S. litura*-infested soybean plants treated with silica NPs over plants treated with water (likelihood ratio *χ*^2^_1_ = 5.62, *P* = 0.018, Fig. 5). There was no difference in preference due to sex (*χ*^2^_1_ = 0.15, *P* = 0.700, Fig. 5). The amount of time taken for a rove beetle to choose one of the two plants was not affected by treatment, sex or season (treatment: *χ*^2^_1_ = 2.81, *P* = 0.094; sex: *χ*^2^_1_ = 0.60, *P* = 0.439; season: *χ*^2^_1_ = 1.57, *P* = 0.210).

### Effects of silica NPs on the germination and growth of *V. faba* seedlings

**Figure 6:**
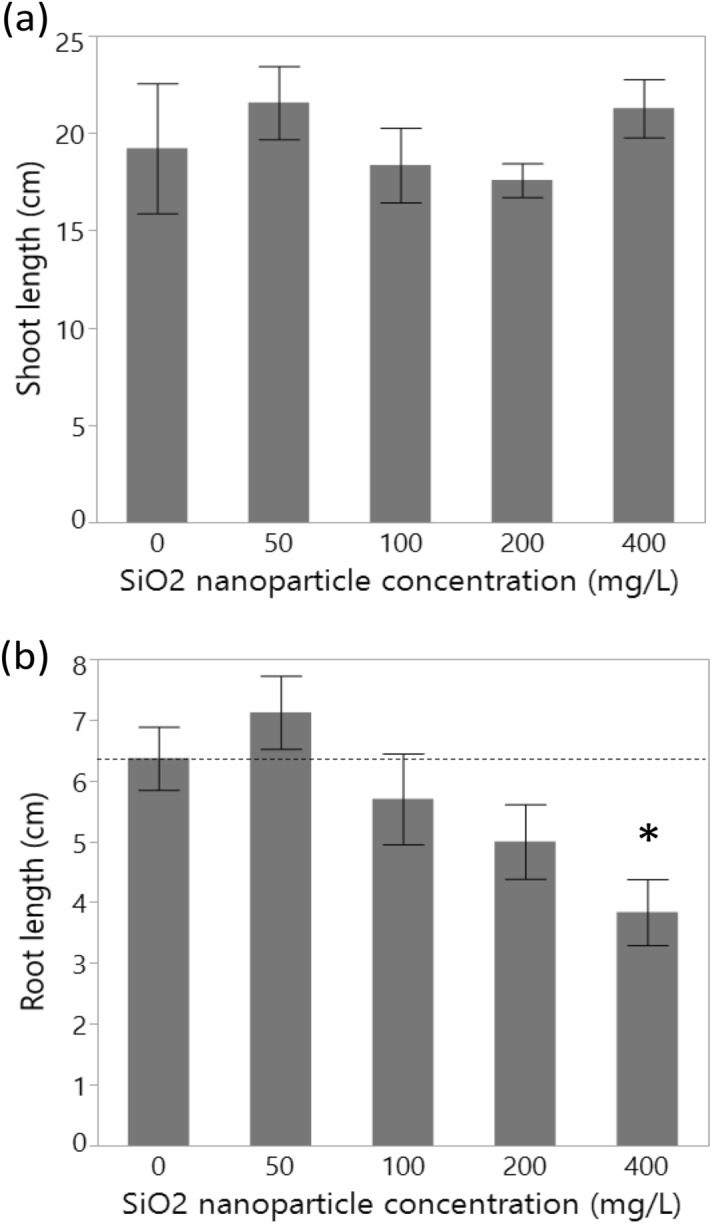
Concentration-dependent effects of silica (SiO_2_) nanoparticles on (**a**) shoot and (**b**) root lengths of faba bean (*Vicia faba*) seedlings. An asterisk indicates a significant difference from the control.

The tested concentrations of silica NPs did not affect germination percentage (*χ*^2^_4_ = 1.21, *P* = 0.877), mean germination time (likelihood ratio *χ*^2^_4_ = 7.05, *P* = 0.133), shoot length (*χ*^2^_4_ = 5.10, *P* = 0.277, Fig. 6a) or SVI (*χ*^2^_4_ = 2.58, *P* = 0.631). However, root length differed significantly among silica NP concentrations (*χ*^2^_4_ = 13.02, *P* = 0.011) and was shorter at 400 mg/L compared to the control treatment (Fig. 6b).

## Discussion

### Pest reduction

This study demonstrated that silica NPs were more effective in controlling the cowpea aphid *A. craccivora* than the other two insect pests, the American serpentine leafminer *L. trifolii* and the cotton leafworm *S. littoralis*. We observed that aphids dropped from the plants immediately after spraying silica NPs. *L. trifolii*, which feeds internally, was less affected at low silica NP concentrations than externally feeding *A. craccivora*. Reductions in *A. craccivora* and *L. trifolii* populations increased with higher concentrations of silica NPs. This is in line with previous studies on the effects of silica NPs on the tomato leafminer *Tuta absoluta*^73^ (greenhouse conditions). Reduction in *S. littoralis* populations also increased with concentrations of silica NPs. This result is consistent with a laboratory study on *S. littoralis*^74^, as well as previous studies that have shown that *S. litura* is effectively controlled by silica NPs (especially when in the hydrophobic or lipophilic form)^75^, and that *S. littoralis* is controlled by silica NPs on tomato plants in a semi-field condition^76^. The higher pest reduction on faba bean is likely due to a higher realized dose of silica NPs per leaf area because of the smaller size of the faba bean plant compared to soybean.

### Attraction and reduction of predators

Our laboratory experiment confirmed that the predatory rove beetle *P. fuscipes* was attracted to volatiles released by infested soybean plants that had been treated with silica NPs. This is the first study to provide evidence that the volatile emitted from infested plants treated with silica NPs is more attractive to predators. This is in contrast to the field test of silica NPs on sugar beet infested by *S. littoralis*, the same pest as in this study, which does not increase the populations of predators such as *Chrysoperla* and *Coccinella* spp.^14^, suggesting that the attractive effect of silica NPs on predators depends on plant species. Our field experiments showed a short-term increase in predators on the soybean infested by a chewing insect but not on the faba bean infested by insects with other feeding types. That anti-herbivore hormonal defenses are induced in Si-treated plants infested with chewing insects (e.g. leafworms) but not fluid feeders (e.g. aphids) is in line with a general trend found in a meta analysis^19^. Because cheweing insects tend to elicit the jasmonic acid signaling cascade while sap feeders induce the salicylic acid pathway^77^, this suggests that chewing insects specifically activate the interaction between Si and jasmonate^19^. Chewing insects also induce more groups of volatiles and in higher magnitudes than phloem feeders^77^, thereby innately tend to allow plants to attract more natural enemies than other feeding guilds.

The role of bulk (non-nano) silicon in attracting natural enemies by enhancing plant hormonal signaling has received recent interest in its applications in plant protection and agriculture^30–33^ (Supplementary Table S2). The application of silicon can enhance the natural defense system of plants by leading to the increased production of flavonoids and phenolic acids^78^ and by promoting jasmonate-mediated defenses^27^ in response to herbivory^79^. Similar effects are expected for nanoscale silica (e.g. silica NPs). In rice, supplemental silicon added to roots for 6–8 weeks altered the profile of HIPVs, subsequently attracting parasitoids of the insects infesting the plant^34^. Similarly, applying silicon to cucumber roots four and seven weeks after potting attracted a predator (*D. bellulus*) of the insect pest *H. armigera* that had infested the cucumber^36^. In both cases, silicon was applied to the roots for an extended period of time before testing the attractiveness of the plants to natural enemies, indicating that bulk silicon does not immediately affect HIPVs when taken in from the root. In our study, applying silica nanoparticles directly to the leaves (with minimum residual silicon in the soil) likely enabled the plants to absorb the silicon molecules at a faster rate. Repeated applications may be necessary to sustain their effects over longer periods of time. It also remains to be established whether silica NP treatments simply augment or modify the profile of the HIPVs emitted by infested soybean plants.

Although the chemical composition of induced resistance within soybean leaves (non-volatiles) by *S. litura* feeding is known^80^, no data are available on the emitted volatiles and compounds essential for the attraction of natural enemies in the soybean-*Spodoptera* system. There is variation in the release of volatiles; while some plants release similar blends of volatiles in response to attacks by different chewing insects^81^, other plants release distinct blends of volatiles in response to differences in the conditions of herbivores^82,83^. Additionally, the relative abundance of salivary proteins that induce plant defense varies even between the strains of an herbivore species^84^. On the other hand, there is also variation in the reception of volatiles; generalist natural enemies do not discriminate among plant volatiles induced by different herbivore species^84–87^. Therefore, generalist rove beetles likely respond similarly to both *S. litura* and *S. littoralis* feeding on the same plant species (soybean).

### Effects on *V. faba* germination and seedling growth

The growth of *V. faba* seedlings was only inhibited by silica NPs at the highest concentration studied (400 mg/L). Silica NPs were likely aggregated at this concentration, resulting in obstruction of the air and water supply through the seed coat, which requires further study. In terms of genotoxicity, however, chromosomal aberrations can be observed in *V. faba* seedlings exposed to silica NPs at much lower concentrations (i.e. 75 mg/L)^49^. The effects of silica NPs on plant growth may be positive^38–43^, non-significant^44,45^ or negative^45–48^ (Supplementary Table S3). These studies also address the optimal concentration of silica NPs required for a positive effect^88^, the alleviation of stress^89^, and the species-dependent effects of silica NPs on plants. Differences in the effects of silica NPs may also be due to other factors such as differences in plants [seed size, seed coat tissue, plant taxonomic groups (monocots or dicots) or even varieties], the nature of growing media (soil, peat moss, agar, etc.), NP property (the size, shape, pH, and aggregation)^90,91^, alongside exposure time to and application method (direct or indirect) of NPs (Supplementary Table S3). Since we washed the seeds to remove residual silica NPs after a day, the exposure time was shorter than several other studies and may have minimized any negative or positive effects.

In summary, this study suggests that silica NPs kill or repel pest insects and predators, directly or indirectly via feeding on treated resources (plants or prey), and contribute to the swift, short-term attraction of predators mediated by the infested plant (soybean). Applying silica NPs at low concentrations of 50–75 mg/L to control insect pests and attract predators on faba bean and soybean will reduce the risks of inhibiting plant growth or critically reducing predator populations.

## Supplementary Information

Supplementary Information.
