# Publisher Correction: Silica nanoparticles as pesticide against insects of different feeding types and their non-target attraction of predators

**DOI:** 10.1038/s41598-021-95389-6

**Published:** 2021-07-30

**Authors:** Ahmed F. Thabet, Hessien A. Boraei, Ola A. Galal, Magdy F. M. El-Samahy, Kareem M. Mousa, Yao Z. Zhang, Midori Tuda, Eman A. Helmy, Jian Wen, Tsubasa Nozaki

**Affiliations:** 1 Economic Entomology Department, Faculty of Agriculture, Kafrelsheikh University, Kafr El-sheikh, Egypt; 2 Genetics Department, Faculty of Agriculture, Kafrelsheikh University, Kafr El-sheikh, Egypt; 3 Field Crop Pests Research Department, Plant Protection Research Institute, Agricultural Research Center, Sakha, Kafr El-sheikh, Egypt; 4 Laboratory of Insect Natural Enemies, Institute of Biological Control, Department of Bioresource Sciences, Faculty of Agriculture, Kyushu University, Fukuoka, 819-0395 Japan; 5 Regional Centre for Mycology and Biotechnology (RCMB), Al-Azhar University, Cairo, Egypt; 6 Entomological Laboratory, Graduate School of Bioresource and Bioenvironmental Sciences, Kyushu University, Fukuoka, Japan; 7 The Kyushu University Museum, Fukuoka, Japan

Correction to: *Scientific Reports* 10.1038/s41598-021-93518-9, published online 14 July 2021

In the original version of this Article Ahmed F. Thabet and Hessien A. Boraei were incorrectly affiliated with ‘The Kyushu University Museum, Fukuoka, Japan’. The correct affiliations are listed below.

Affiliation 1:

Economic Entomology Department, Faculty of Agriculture, Kafrelsheikh University, Kafr El-sheikh, Egypt

Ahmed F. Thabet and Hessien A. Boraei

Affiliation 2:

Genetics Department, Faculty of Agriculture, Kafrelsheikh University, Kafr El-sheikh, Egypt

Ahmed F. Thabet

Affiliation 3:

Field Crop Pests Research Department, Plant Protection Research Institute, Agricultural Research Center, Sakha, Kafr El-sheikh, Egypt

Ahmed F. Thabet

Affiliation 4:

Laboratory of Insect Natural Enemies, Institute of Biological Control, Department of Bioresource Sciences, Faculty of Agriculture, Kyushu University, Fukuoka, 819-0395, Japan

Ahmed F. Thabet

In addition, Ahmed F. Thabet and Hessien A. Boraei were incorrectly listed as equally contributing authors.

Finally, Hessien A. Boraei is deceased.

The original Article has been corrected.

