# Supplementary material for: Silica nanoparticles as pesticide against insects of different feeding types and their non-target attraction of predators
Source: Sci Rep. 2021 Jul 14;11:14484. doi: 10.1038/s41598-021-93518-9 (PMC8280210; doi:10.1038/s41598-021-93518-9)
Supplement: Supplementary file 1 — Supplementary Information. [file 41598_2021_93518_MOESM1_ESM.docx]

Silica nanoparticles as pesticide against insects of different feeding types and their non-target attraction of predators

A.F. Thabet ^1,2,3,4*^, H. A. Boraei ^1^, O.A. Galal ^2^, M.F.M. El-Samahy ^3^, K. M. Mousa ^1,4^, Y. Z. Zhang ^4^, M. Tuda ^4*^, E. A. Helmy ^4,5^, J. Wen ^4^, T. Nozaki ^6,7^

^1^ Economic Entomology Department, Faculty of Agriculture, Kafrelsheikh University, Kafr Elsheikh, Egypt

^2^ Genetics Department, Faculty of Agriculture, Kafrelsheikh University, Kafr Elsheikh, Egypt

^3^ Field Crop Pests Research Department, Plant Protection Research Institute, Agricultural Research Center, Sakha, Kafr Elsheikh, Egypt

^4^ Laboratory of Insect Natural Enemies, Institute of Biological Control, Department of Bioresource Sciences, Faculty of Agriculture, Kyushu University, Fukuoka, Japan

^5^ Regional Centre for Mycology and Biotechnology (RCMB), Al–Azhar University, Cairo, Egypt

^6^ Entomological Laboratory, Graduate School of Bioresource and Bioenvironmental Sciences, Kyushu University, Fukuoka, Japan

^7^ The Kyushu University Museum, Fukuoka, Japan

**Supplementary Table S1.** Silica (SiO_2_) nanoparticle effects on insects. Non-exhaustive list of studies.

| Order | Species | Plant | Feeding type | Setting | SiO_2_ conc. | Developmental stage | Exposure time | Survival rate | Weight | Reference |
| --- | --- | --- | --- | --- | --- | --- | --- | --- | --- | --- |
| Coleoptera | *Sitophilus oryzae* | rice | internal seed feeder | lab. | 0.5, 1, 2 g/kg | adult | 1, 2, 4, 7, 14 d | − @ 2 g/kg |  | Debnath et al. 2011 |
|  | *Sitophilus granarius* | wheat | internal seed feeder | lab. | 1, 2, 3 g/kg | larva, adult | 1, 2, 4, 7, 14 d | − @ 3 g/kg  (larva, adult) |  | Rouhani et al. 2019 |
|  | *Callosobruchus maculatus* | cowpea | internal seed feeder | lab. | 1, 1.5, 2, 2.5 g/kg | larva, adult | 1,2,4,7,14 d | − @ 2.5 g/kg |  | Rouhani et al. 2013 |
|  | *Rhyzopertha dominica* | wheat, barley | internal seed feeder | lab. | 50, 100, 200 and 300 mg/kg | adult | 1, 2, 3, 7 d | − @ 300 mg/kg |  | Ziaee and Ganji 2016 |
|  | *Tribolium confusum* | wheat, barley | internal seed feeder | lab. | 50, 100, 200, 300 mg/kg | adult | 1, 2, 3, 7 d | − @ 300 mg/kg |  | Ziaee and Ganji 2016 |
|  | *Coccinella* spp. | sugar beet | insect predator | field | 15, 30, 45, 60, 75 g/4200 m^2^ | larva, adult | 2, 5, 7, 10, 15 d | − |  | El-Samahy et al. 2015 |
| Diptera | *Liriomyza trifolii* | kidney bean | internal leaf feeder | lab. | 50, 100, 200, 400 mg/L | larva, pupa |  | − @ 400 mg/L (larva) | − @ 50, 100, 400 mg/L (pupa) | Thabet et al. unpublished |
|  | *Liriomyza trifolii* | faba bean | internal leaf feeder | field | 100, 200, 300, 400, 500 mg/L | larva | 1, 3, 5, 7, 10, 15 d | − (number) |  | El-Samahy and Galal, 2012 |
|  | *Liriomyza trifolii* | faba bean | internal leaf feeder | field | 75, 150, 225, 300, 375, 425 mg/L | larva | 1, 3, 5, 7, 10, 15 d | − (number) |  | This study |
| Lepidoptera | *Spodoptera littoralis* | tomato | external chewer | lab. | 100,150, 200, 250, 300, 350 ppm | larva | 15 d | − @ 350 ppm (larva) |  | El-Bendary and El-Helaly, 2013 |
|  | *Spodoptera littoralis* | castor | external chewer | lab. | 250, 500, 1000 ppm | larva |  | − @ 500, 1000 ppm (larva) |  | Ayoub et al. 2017 |
|  | *Spodoptera littoralis* | sugar beet | external chewer | field | 15, 30, 45, 60, 75 g/4200 m^2^ | larva | 2, 5, 7, 10, 15 d | − @ 45, 60, 75 g/4200 m^2^ |  | El-Samahy et al. 2015 |
|  | *Spodoptera littoralis* | soybean | external chewer | field | 75, 150, 225, 300, 375, 425 mg/L | larva | 1, 3, 5, 7, 10, 15 d | − (number) |  | This study |
|  | *Mythimna separata* | wheat | external chewer | lab. | 5% in soil, 0.4% foliar | larva |  | − @ 0.4% foliar | − @ 0.4% foliar | Mousa et al. 2014 |
|  | *Plutella xylostella* | cabbage | external chewer | lab. | 0.125, 0.25, 0.5, 0.75, 1 mg/cm^2^ | larva | 24, 48, 72 h | − @ 1 mg/cm^2^ |  | Shoaib et al. 2018 |
|  | *Plutella xylostella* | cabbage | external chewer | lab. | 100, 200, 400, 800, 1200 mg/L | larva |  | − @ 1200 mg/L |  | Shoaib et al. 2018 |
|  | *Tuta absoluta* | tomato | internal leaf chewer | field | 150, 300, 600 ppm | larva |  | − @ 600 ppm |  | Fouad et al. 2016 |
| Hemiptera | *Lipaphis pseudobrassicae* | rapeseed | external sucker | lab. | 0.025, 0.05, 0.1 mg/cm^2^ | nymph | 12 h | − @ 0.1 mg/cm^2^ |  | Debnath et al. 2010 |
|  | *Aphis craccivora* | faba bean | external sucker | field | 100, 200, 300, 400, 500 mg/L | nymph, adult | 1, 5, 8, 10, 15 d | − (number) |  | El-Samahy and Galal, 2012 |
|  | *Aphis craccivora* | faba bean | external sucker | field | 75, 150, 225, 300, 375, 425 mg/L | nymph, adult | 1, 3, 5, 7, 10, 15 d | − (number) |  | This study |
| Neuroptera | *Chrysoperla carnea* | sugar beet | insect predator | field | 15, 30, 45, 60, 75 g/4200 m^2^ | larva |  | − |  | El-Samahy et al. 2015 |
| Araneae | True spider | sugar beet | insect predator | field | 15, 30, 45, 60, 75 g/4200 m^2^ |  |  | − |  | El-Samahy et al. 2015 |

**Supplementary Table S2.** Silica (SiO_2,_ bulk size) effects on insects. Non-exhaustive list of studies.

| Order | Species | Plant | Feeding type | Setting | SiO_2_ conc. | Developmental stage | Exposure time | Survival rate | Weight | Reference |
| --- | --- | --- | --- | --- | --- | --- | --- | --- | --- | --- |
| Coleoptera | *Sitophilus granarius* | wheat | internal seed feeder | lab. | 0.125, 0.25, 0.5, 1, 2, 3 kg/t | adult | ~ 31 d | − @ 1, 2, 3 kg/t |  | Zakladnoy 2019 |
|  | *Cryptolestes ferrugineus* | wheat | internal seed feeder | lab. | 0.125, 0.25, 0.5, 1, 2, 3 kg/t | adult | ~ 31 d | − @ 1, 2, 3 kg/t |  | Zakladnoy 2019 |
|  | *Oryzaephilus surinamensis* | wheat | internal seed feeder | lab. | 0.125, 0.25, 0.5, 1, 2, 3 kg/t | adult | ~ 31 d | − @ 1, 2, 3 kg/t |  | Zakladnoy 2019 |
| Neuroptera | *Chrysoperla externa* | wheat | insect predator | lab. | 100 ml (1%)/pot | egg, larva, adult | Ca. 10 d | NS (developmental duration) |  | Moraes et al. 2004 |
| Hymenoptera | *Aphidius colemani* | wheat | parasitoid | lab. | 2.5 g (38%)/kg | egg, larva, adult | 8 d | NS (developmental duration, parasitism) |  | Moraes et al. 2004 |
| Lepidoptera | *Chilo suppressalis* | rice | internal chewer | glasshouse | 150, 600 kg/ha | larva | 96 h |  | − @600 kg/ha | Hou, Han 2010 |
|  | *Busseola fusca* | maize, sorghum, wild grasses | internal chewer | greenhouse | 0.5, 1, 1.5, 2 % | larva | 7, 19, 31 d | − @ 1.5% |  | Juma et al. 2015 |
|  | *Eldana saccharina* | sugarcane | internal chewer | shade house | 5000, 10000 kg/ha | larva, pupa |  | − @10000 kg/ha  (larva, pupa) | − @5000 kg/ha (larva, pupa) | Keeping and Meyer 2002 |
| Hemiptera | *Phenacoccus solenopsis* | moss rose | external sucker | field | 0.06% | nymph, adult | 1, 3, 9, 13, 21 d | − @ 13, 21 d |  | Huang et al. 2014 |
|  | *Schizaphis graminum* | wheat | external sucker | lab. | 2.5 g/kg soil, 2.5 g/kg soil+0.5% foliar appl. | nymph, adult |  | − @ soil application |  | Goussain et al.2005 |
|  | *Schizaphis graminum* | wheat | external sucker | lab. | 100 ml (1%)/pot | nymph, adult | 24, 48, 72 h | − (number) |  | Moraes et al. 2004 |
|  | *Myzus persicae* | elegant zinnia | external sucker | lab. | 20 ml/L | nymph, adult | 18 d | NS (nymph, adult), − (fecundity, intrinsic rate of increase) |  | Ranger et al 2009 |

**Supplementary Table S3**. Silica (SiO_2_) nanoparticle effects on plant growth. NS, non-significant effect; +, higher than control; −, lower than control; @, at a concentration and/or a particle size; max, maximum; min, minimum.

| Family | Species | Concentration (g/L) | Size (nm) | Exposure (day) | Germination | Vigor index | Root length | Shoot length | Reference |
| --- | --- | --- | --- | --- | --- | --- | --- | --- | --- |
| Poaceae | *Agropyron elongatum* | 0.005, 0.02, 0.04, 0.06, 0.08 | 10–15 | 14 | + @ .005, .02, .04, .06 |  | + | + @ .005, .06 | Azimi et al. 2014 |
| Poaceae | *Oryza sativa* | 2 | 5–15 | 7 | NS |  | NS | NS | Yang et al. 2015 |
| Poaceae | *Zea mays* | 2 | 5–15 | 5 | NS |  | NS | NS | Yang et al. 2015 |
| Poaceae | *Zea mays* | 0.4, 2, 4 | 10–20 | 1 or 14 | + @ .4, − @ 2, 4 (1 d) |  | + @ .4, − @ 2, 4 (14 d) | + @ .4, − @ 2, 4 (14 d) | Sharifi-Rad et al. 2016 |
| Fabaceae | *Phaseolus vulgaris* | 0.4, 2, 4 | 10–20 | 1 or 14 | + @ .4, − @ 2, 4 (1 d) |  | + @ .4, − @ 2, 4 (14 d) | + @ .4, − @ 2, 4 (14 d) | Sharifi-Rad et al. 2016 |
| Fabaceae | *Lens culinaris* | 0.025, 0.05, 0.075, 0.1, 0.2, 0.3 |  | 1 | − @ .2, .3 | NS | − @ .2, .3 | − @ .2, .3 | Khan and Ansari 2018 |
| Fabaceae | *Lens culinaris* | 0.06 | 20–30 | 10 | + under salinity |  | + under salinity | + under salinity | Sabaghnia and Janmohammadi 2014 |
| Fabaceae | *Vicia faba* | 0.06, 0.12, 0.18 | 40 |  | + under salinity |  |  | +@ .06, .12 under salinity | Qados and Moftah 2015 |
| Fabaceae | *Vicia faba* | 0.09, 0.18 |  | 65 | NS |  | + |  | Roohizadeh et al. 2015 |
| Fabaceae | *Vicia faba* | 0.025, 0.050, 0.075 | 119.1 ± 2.8 | 1 | NS | NS | NS | NS | Thabet et al. 2019 |
| Fabaceae | *Vicia faba* | 0.05, 0.1, 0.2, 0.4 | 19.6 ± 5.8 | 1 | NS | NS | − @ 0.4 | NS | This study |
| Cucurbitaceae | *Cucurbita pepo* | 1.5, 3,4.5, 6, 7.5 | 10 | 15 | + under salinity | + under salinity | + under salinity | + under salinity | Siddiqui et al. 2014 |
| Cucurbitaceae | *Cucumis sativus* | 0.1, 0.2, 0.3, 0.4 | 10 | .17 | + @ .2 | + @ .2 | + | + | Alsaeedi et al. 2019 |
| Amaryllidaceae | *Allium cepa* | 0.54, 0.81, 1.2, 1.82 | 22 | 4 | − @ .54, .81, 1.82 |  | [− @ .54](mailto:-@.54), 1.82 |  | Silva and Monteiro 2017 |
| Amaryllidaceae | *Allium cepa* | 0.54, 0.81, 1.2, 1.82 | 12 | 4 | − |  | − |  | Silva and Monteiro 2017 |
| Amaryllidaceae | *Allium cepa* | 0.54, 0.81, 1.2, 1.82 | 7 | 4 | −@ .54, .81, 1.82 |  | − @ .81, 1.2, 1.82 |  | Silva and Monteiro 2017 |
| Solanaceae | *Lycopersicum esculentum* | 2, 4, 6, 8, 10, 12, 14 | 12 | 10 | max @ 8 | max @ 8 |  |  | Siddiqui and Al-Wahibi 2014 |
| Brassicaceae | *Arabidopsis thaliana* | 0.4, 2, 4 | 42.8 ± 3.9 | 18 | NS |  | + @ .4, − @ 2, 4 |  | Lee et al. 2010 |
| Lamiaceae | *Hyssopus officinalis* | 0.4, 2, 4 | 10–20 | 1 or 14 | + @.4, − @ 2, 4 (1 d) |  | + @ .4, − @ 2, 4 (14 d) | + @ .4, − @ 2, 4 (14 d) | Sharifi-Rad et al. 2016 |
| Ranunculaceae | *Nigella sativa* | 0.4, 2, 4 | 10–20 | 1 or 14 | + @.4, − @ 2, 4 (1 d) |  | + @ .4, − @ 2, 4 (14 d) | + @ .4, − @ 2, 4 (14 d) | Sharifi-Rad et al. 2016 |
| Amaranthaceae | *Amaranthus retroflexus* | 0.4, 2, 4 | 10–20 | 1 or 14 | − (1 d) |  | − @ 2, 4 (14 d) | −, min @ 4 (14 d) | Sharifi-Rad et al. 2016 |
| Asteraceae | *Taraxacum officinale* | 0.4, 2, 4 | 10–20 | 1 or 14 | − (1 d) |  | −, min @ 4 (14 d) | −, min @ 4 (14 d) | Sharifi-Rad et al. 2016 |

**References**

Alsaeedi AH, Elgarawany MM, El-Ramady H, Alshaal T, AL-Otaibi AOA (2019) Application of silica nanoparticles induces seed germination and growth of cucumber (*Cucumis sativus*). Met Env Arid Land Agric Sci 28, 57–68.

Ayoub HA, Khairy M, Rashwan FA, Abdel-Hafez HF (2017) Synthesis and characterization of silica nanostructures for cotton leaf worm control. J Nanostruct Chem 7,91–100.

Azimi R, Borzelabad MJ, Feizi H, Azimi A (2014) Interaction of SiO_2_ nanoparticles with seed prechilling on germination and early seedling growth of tall wheatgrass (*Agropyron elongatum* L.). Pol J Chem Tech 16, 25–29.

Debnath N, Das S, Brahmachary RL, Chandra R, Sudan S, Goswami A (2010) Entomotoxicity assay of silica, zinc oxide, titanium dioxide, aluminium oxide nanoparticles on *Lipaphis pseudobrassicae*. ICANN 2009. In: Giri PK, Goswami DK, Perumal A, Chattopadhyay A (eds) Book Series: AIP Conference Proceedings 1276, 307.

Debnath N, Das S, Seth D, Chandra R, Bhattacharya SC, Goswami A (2011) Entomotoxic effect of silica nanoparticles against *Sitophilus oryzae* (L.). J Pest Sci 84, 99–105.

El-Bendary HM, El-Helaly AA (2013) First record nanotechnology in agricultural: Silica nano-particles a potential new insecticide for pest control. Applied Science Reports 4, 241–246.

El-Samahy MFM, Galal OA (2012) Evaluation of silica nanoparticles as a new approach to control faba bean (*Vicia faba* L.) insects and its genotoxic effect on M2 plants. Egypt. J Agric Res 90, 869–888.

El-Samahy MFM, Khafagy IF, El- Ghobary AMA (2015) Efficiency of silica nanoparticles, two bioinsecticides, peppermint extract and insecticide in controlling cotton leafworm, *Spodoptera littoralis* Boisd. and their effects on some associated natural enemies in sugar beet fields. J Plant Prot and Path, Mansoura Univ 9, 1221–1230.

Fouad HA, El-Gepaly HMH, Fouad OA (2016) Nanosilica and jasmonic acid as alternative methods for control *Tuta absoluta* (Meyrick) in tomato crop under field conditions. Arch Phytopathol Pflanzenschutz 49, 362–370.

Goussain MM, Prado E, Moraes JC (2005) Effect of silicon applied to wheat plants on the biology and probing behaviour of the greenbug *Schizaphis graminum* (Rond.) (Hemiptera: Aphididae). Neotrop Entomol 34, 807–813.

Hou M, Han Y (2010) Silicon-mediated rice plant resistance to the Asiatic rice borer (Lepidoptera: Crambidae): effects of silicon amendment and rice varietal resistance, J Econ Entomol 103, 1412–1419.

Huang J, Zhang J, Wang D, Zhang L, Xu Y, Li M (2014) Do organic silicon and imidacloprid synergistically induce toxicity to the new invasive mealybug *Phenacoccus solenopsis* Tinsley on *Portulaca grandiflora* plants? Turk J Agric For 38, 207–213.

Juma G, Ahuya P, Ong'amo G, Le Ru B, Magoma G, Silvain J, Calatayud P (2015). Influence of plant silicon in *Busseola fusca* (Lepidoptera: Noctuidae) larvae – Poaceae interactions. Bull Entomol Res 105, 253–258.

Keeping MG, Meyer JH (2002) Calcium silicate enhances resistance of sugarcane to the African stalk borer *Eldana saccharina* Walker (Lepidoptera: Pyralidae). Agric Forest Entomol 4, 265–274.

Khan Z, Ansari MYK (2018) Impact of engineered Si nanoparticles on seed germination, vigour index and genotoxicity assessment via DNA damage of root tip cells in *Lens culinaris*. J Plant Biochem Physiol 6, 5243–5246.

Lee CW, Mahendra S, Zodrow K, Li D, Tsai YC, Braam J, Alvarez PJJ (2010) Developmental phytotoxicity of metal oxide nanoparticles to *Arabidopsis thaliana*. Environ Toxicol Chem 29, 669–675.

Moraes JC, Goussain MM, Basagli MAB, Carvalho GA, Ecole CC, Sampaio MV (2004) Silicon influence on the tritrophic interaction: wheat plants, the greenbug *Schizaphis graminum* (Rondani) (Hemiptera: Aphididae), and its natural enemies, *Chrysoperla externa* (Hagen) (Neuroptera: Chrysopidae) and *Aphidius colemani* Viereck (Hymenoptera: Aphidiidae). Neotrop Entomol 33, 619–624.

Mousa KM, Elsharkawy MM, Khodeir IA, El-Dakhakhni TN, Youssef AE (2014) Growth perturbation, abnormalities and mortality of oriental armyworm *Mythimna separata* (Walker) (Lepidoptera: Noctuidae) caused by silica nanoparticles and *Bacillus thuringiensis* toxin. Egypt J Biol Pest Control 24, 283–287.

Qados AMSA, Moftah AE (2015) Influence of silicon and nanosilicon on germination, growth and yield of faba bean (*Vicia faba* L.) under salt stress conditions. Am J Exp Agric 5, 509–524.

Ranger CM, Singh AP, Frantz JM, Canas L, Locke JC, Reding ME, Vorsa N (2009) Influence of silicon on resistance of *Zinnia elegans* to *Myzus persicae* (Hemiptera: Aphididae). Environ Entomol 38, 129–136.

Roohizadeh G, Majd A, Arbabian S (2015) The effect of sodium silicate and silica nanoparticles on seed germination and some of growth indices in the *Vicia faba* L. Trop Plant Res 2, 85–89.

Rouhani M, Samih MA, Kalantari S (2013) Insecticidal effect of silica and silver nanoparticles on the cowpea seed beetle, *Callosobruchus maculatus* F. (Col.: Bruchidae). J Entomol Res 4, 297–305.

Rouhani M, Samih MA, Zarabi M, Beiki K, Gorji M, Aminizadeh MR (2019) Synthesis and entomotoxicity assay of zinc and silica nanoparticles against *Sitophilus granarius* (Coleoptera: Curculionidae). J Plant Prot Res 59, 26–31.

Sabaghnia N, Janmohammadi M (2014) Effect of nanosilicon particles application on salinity tolerance in early growth of some lentil genotypes. Ann. UMCS Biol 69, 39–55.

Sharifi-Rad J, Sharifi-Rad M, Teixeira da Silva JA (2016) Morphological, physiological and biochemical responses of crops (*Zea mays* L., *Phaseolus vulgaris* L.), medicinal plants (*Hyssopus officinalis* L., *Nigella sativa* L.), and weeds (*Amaranthus retroflexus* L., *Taraxacum officinale* F. H. Wigg) exposed to SiO_2_ nanoparticles. J Agric Sci Technol 18, 1027–1040.

Shoaiba A, Elabasya A, Waqasa M, Lina L, Chenga X, Zhanga Q, Shia Z (2018) Entomotoxic effect of silicon dioxide nanoparticles on *Plutella xylostella* (L.) (Lepidoptera: Plutellidae) under laboratory conditions. Toxicol Environ Chem 100, 1–12.

Siddiqui MH, Al-Whaibi MH, Faisal M, Al Sahli AA (2014) Nanosilicon dioxide mitigates the adverse effects of salt stress on *Cucurbita pepo* L. Environ Toxicol Chem 33, 2429–2437.

Silva GH, Monteiro RT (2017) Toxicity assessment of silica nanoparticles on *Allium cepa*. Ecotox Environ Contam 12, 25–31.

Thabet AF, Galal OA, El‑Samahy MFM, Tuda M (2019) Higher toxicity of nano‑scale TiO2 and dose‑dependent genotoxicity of nano‑scale SiO2 on the cytology and seedling development of broad bean *Vicia faba*. SN Applied Sciences 1, 956.

Yang Z, Chen J, Dou R, Gao X, Mao C, Wang L (2015) Assessment of the phytotoxicity of metal oxide nanoparticles on two crop plants, maize (*Zea mays* L.) and rice (*Oryza sativa* L.). Int J Environ Res Public Health 12, 15100–15109.

Zakladnoy GA (2019) Response of insect pests of stored grain to silicon dioxide treatment. Entmol Rev 99, 1125–1127.

Ziaee M, Ganji Z (2016) Insecticidal efficacy of silica nanoparticles against *Rhyzopertha dominica* F. and *Tribolium confusum* Jacquelin du Val. J Plant Prot Res 56, 250–256.
